# Prospective analysis of breast masses using the combined score for quantitative ultrasonography parameters

**DOI:** 10.1038/s41598-022-19971-2

**Published:** 2022-09-28

**Authors:** Eun Ji Lee, Yun-Woo Chang

**Affiliations:** Department of Radiology, Soonchunhyang University Hospital, 59 Daesakwan-Ro, Yongsan-Ku, Seoul, 04401 South Korea

**Keywords:** Oncology, Diseases, Cancer

## Abstract

To investigate the diagnostic value of combined SWE, SMI, and B-mode US scores for distinguishing between benign and malignant masses. A total of 450 breast masses that underwent US-guided core needle biopsies were prospectively enrolled. The breast masses were assessed based on the BI-RADS and quantitative SWE and SMI parameters. The SWEmax, SWEratio, and SMI_VI_ cutoff value were determined using Youden’s index by comparison to the pathological results. The BI-RADS categories were scored on a scale from 1 to 5, and SWEmax, SWEratio, and SMI_VI_ were dichotomized based on each cutoff values (0 or 1). The combined scores (1 to 8) were calculated as the sum of the BI-RADS score and the quantitative scores and compared to the pathologic results using AUROC analysis. The cutoff values were 52.25 kPa for SWEmax, 5.03 for SWEratio, and 2.15% for SMI_VI_. In AUROC, the combined scores showed significantly better diagnostic performance compared to BI-RADS alone (*p* < 0.001). The combined score showed significantly increased than BI-RADS alone in specificity (*p* < 0.001) and accuracy (*p* < 0.001), but a sensitivity decreased without significance (*p* = 0.082). When a combined score cutoff value of 4 was used, the false negative rate was 2.7%. Using the combined score, 76.4% of the C4a lesions were considered benign also pathologically diagnosed as benign. The combined scores showed improved diagnostic performance in differentiating between benign and malignant breast masses, which could be helpful for determining a breast biopsy eligibility.

## Introduction

The evaluation of breast masses on ultrasonography (US) is based on B-mode US, and breast masses are categorized according to the Breast Imaging Reporting and Database System (BI-RADS)^1^. Although B-mode US based on the BI-RADS assessment category has a high sensitivity in differentiating benign from malignant breast masses, the relatively wide range and low specificity results in a high false positive rate, leading to unnecessary biopsies^2–4^. In addition to B-mode US, supplementary techniques have been developed to compensate for the low specificity of B-mode US by adding information on tissue elasticity and vascularity. Shear wave elastography (SWE) is a technique that evaluates tissue stiffness by inducing a push pulse into the tissue and measuring the speed of the propagating shear waves within the tissue^5^. Superb microvascular imaging (SMI) is a new technique that can separate and detect slow blood flow signals, which are removed along with overlaying tissue motion artifacts in conventional Doppler imaging^6^. A recent meta-analysis of 21 studies on supersonic shear imaging reported that the combination of SWE and the B-mode significantly increased the pooled specificity from 0.61 to 0.85 compared to B-mode alone for evaluating breast masses, resulting in better diagnostic performance^7^. This suggests that adding SWE to B-mode US may be a clinically acceptable practice. A few studies reported that when combined with elastography, BI-RADS category 4a lesions were downgraded and category 3 lesions were upgraded^5,8,9^. In addition, there are increasing reports that the combined use of SMI to B-mode US could improve diagnostic performance compared to B-mode alone by increasing in specificity^6,10–17^. Lee et al.^18^. reported that combining all quantitative values for SWEmax, SWEratio and SMI_VI_ with B-mode US improved the diagnostic performance in differentiating between benign and malignant lesions compared to B-mode alone. The purpose of this study was to investigate the diagnostic value of the combined use of B-mode US with the quantitative SWE and SWI_VI_ parameters for differentiating between benign and malignant breast masses in prospectively enrolled patients using a combined scoring system that was easy to apply.

## Materials and methods

### Study participants

**Figure 1:**
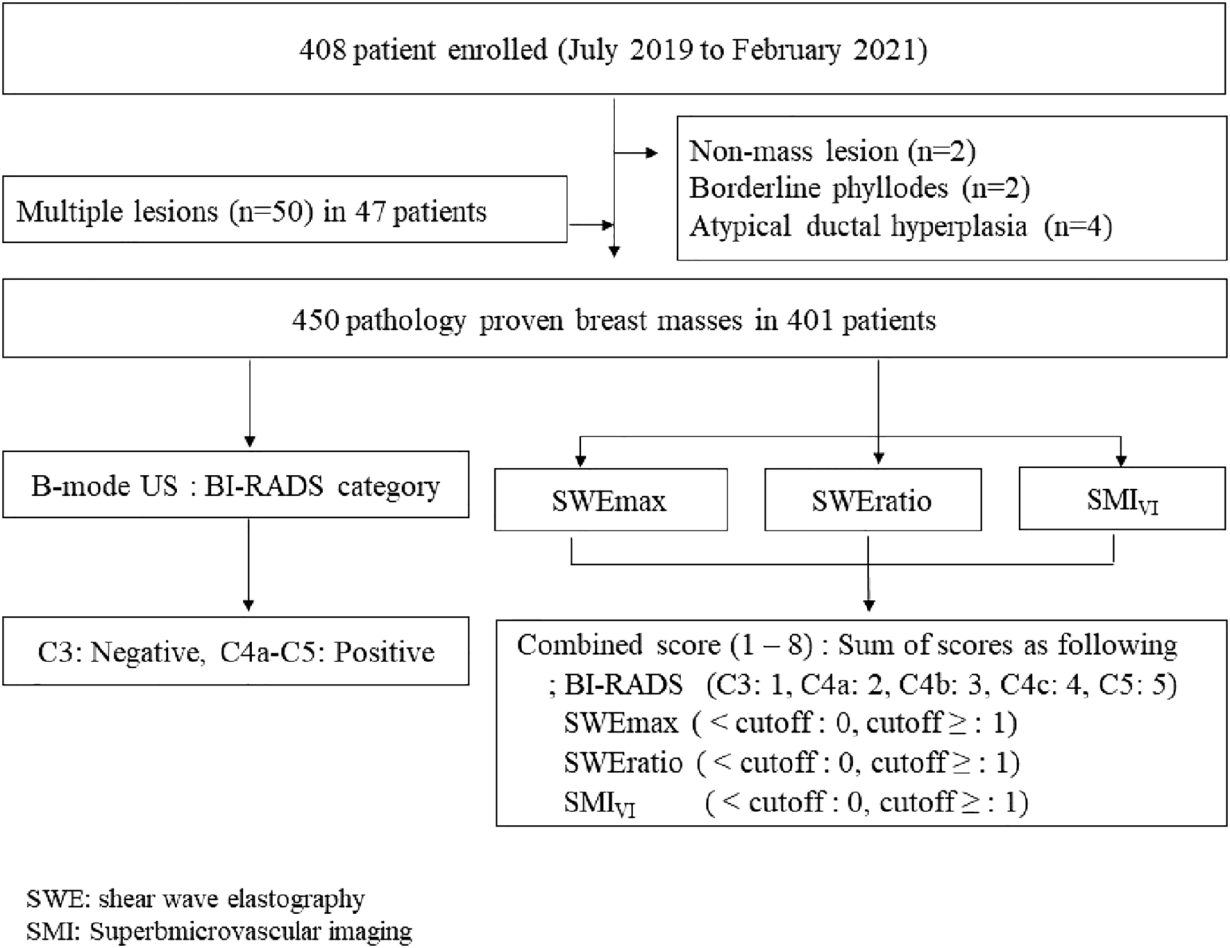
Study population.

This prospective study was approved by our Institutional Review Board for Ethical Issues in Clinical Research (Soonchunhyang University Seoul Hospital Institutional review board No. 2019-05-013) and complied with the Declaration of Helsinki. The written informed consent was obtained from all participants before examination. From July 2019 to February 2021, adult women older than 19 years who received US-guided core needle biopsies and breast US including B-mode US, shear wave elastography (SWE), and superb microvascular imaging (SMI) were enrolled in the study. The B-mode US, SWE and SMI examinations were performed on the same day as the biopsy or one month prior to the biopsy. A total of 408 patients were enrolled. Those with non-mass lesions (n = 2) and borderline pathology including borderline phyllodes tumor (n = 2) and atypical ductal hyperplasia (n = 4) were excluded. In 47 patients, fifty lesions of multiple core biopsies were included. Finally, a total of 450 lesions of 401 patients were analyzed (Fig. 1). All participants were women and the mean age was 45.8 ± 12.1 years (range, 20–84 years).

### Ultrasonography examination

All US examinations were performed by one of two board-certificated radiologists who had 19 and 4 years of experience in breast imaging and both had 2 years of experience in SWE and SMI. Breast US examinations were performed using the US equipment of the same model of US systems (Aplio 800, Canon Medical Systems Corporation, Tokyo, Japan) equipped with a 7- to 18-MHz multi-frequency ultra-wide band linear array transducer. Breast US examinations were performed with conventional B-mode US followed by SWE and SMI. SWE and SMI were obtained using the same depth, focus, and time gain used in B-mode US. In B-mode US, two orthogonal planes (transverse and longitudinal planes) of each lesion were obtained and the maximum diameter of the lesion was measured. Shear wave elastography was performed with ROI box including the entire lesion and normal fat or glandular tissue in one-shot mode. Adequate SWE quality was evaluated by real-time US by checking the B-mode US, color map, variance map, and propagation map that were simultaneously displayed by a split-screen view of a single screen. At least tow or more SWE images per lesion were obtained, and an image of the best quality of shear wave propagation showing a homogeneous variance map was selected for the measurement of quantitative parameters. The maximum elasticity of the SWE image was set to display 120–180 kPa. Quantitative elasticity values were measured by applying a 2-mm-sized circular region of interest (ROI) over the stiffest portion of the lesion or the boundary of the lesion and by applying another circular ROI in the adjacent normal fat tissue. By setting the ROIs, quantitative elasticity values including the maximum elasticity (SWEmax) and the elasticity ratio between the lesion and subcutaneous fat tissue (SWEratio) were automatically calculated by the US system.

In SMI, the vascular index (SMI_VI_) was automatically calculated for the quantitative measurement of mass vascularity. SMI_VI_ was measured by the US system by drawing a manual ROI along the margin of the mass at the plane with the richest Doppler signal within the mass. The image parameters for SMI were velocity scale, 2.5 cm/s, dynamic range, 21–40 dB, and frame rate, 13–52 frames/s. SMI_VI_ was also measured two or more times for each breast lesion. The total breast US data acquisition time took about 3–5 min per lesion.

### Image analysis

The selection of the representative images of breast US data including B-mode and quantitative SWE parameters (SWEmax, and SWEratio) and SMI (SMI_VI_) was performed by the radiologist who performed the breast US. All breast lesions were assessed by BI-RADS based on B-mode US and classified as category C3 (probably benign: 2% likelihood of malignancy or less), C4a (low suspicion of malignancy; greater than 2% to 10% likelihood of malignancy), C4b (moderate suspicion of malignancy; greater than 10% to 50% likelihood of malignancy), C4c (high suspicion of malignancy; greater than 50% to 95% likelihood of malignancy), and C5 (highly suggest of malignancy; 95% or greater likelihood of malignancy). Assessment category 3 (probably benign) suggests a likelihood of malignancy with the defined < 2% for which short-interval (6-month) follow-up sonography and then periodic sonographic surveillance may represent appropriate management. Category 4 (suspicious abnormality) is reserved for finding that does not have the classic appearance of malignancy but is sufficiently suspicious to justify a recommendation for biopsy. The ceiling for C3 assessment is a 2% likelihood of malignancy, and the floor for category 5 assessment is 95%, so category 4 assessment covers the wide range of likelihood of malignancy in between. Thus, almost all recommendations for breast interventional procedures will come from assessments category 4 or 5.

Regarding B-mode US analysis, BI-RADS category C3 masses were considered benign, and BI-RADS category C4a and higher masses were considered a positive result for malignancy.

To analyze the diagnostic performance of the combined B-mode US and SWE and SMI quantitative parameters, the combined score was used. For B-mode US, the BI-RADS categories were scored on a scale of 1 to 5 (C3; 1, C4a; 2, C4b; 3, C4c; 4, and C5; 5). For SWE and SMI, the SWEmax, SWEratio, and SMI_VI_ cutoff values were determined using the Youden index by comparison to the pathological results, and each value was scored as 0 when less than the cutoff value, and 1 for higher than the cutoff value. The combined score was calculated as the sum of the BI-RADS score and each quantitative parameter score, ranging from 1 to 8.

### Statistical analysis

The pathologic results from the US-guided core needle biopsy were used as the reference standard for direct comparison with quantitative parameters of the mass. The SWEmax, SWEratio, and SMI_VI_ cutoff values used to optimally differentiate between benign and malignant masses were determined by a receiver operating characteristic (ROC) curve analysis using Youden’s index^19^. B-mode BI-RADS category and combined scores were compared to the pathology results. For the statistical analysis of the diagnostic performance of BI-RADS alone, the BI-RADS categories were divided into two groups; those with negative results were classified as C3 and those with positive results were C4a and above. The diagnostic performance of B-mode US alone based on BI-RADS assessment, combined BI-RADS, and all quantitative SWE and SMI parameter scores were determined by area under the ROC (AUROC) curve analysis. The AUROC values, sensitivity, specificity, accuracy, PPV, and NPV were compared to BI-RADS alone and combined score. Statistical analyses were performed using the Statistical Package for the Social Sciences (SPSS) version 20.0 (IBM Corp.) and Rex 3.1.2 version (rexsoft.org). *P*-values of less than 0.05 were considered statistically significant.

### Image evaluation in validation cohort

From March 2021 to April 2022, the combination score 4 was applied as a cut value to 524 masses of 461 patients who performed sonography-guided core needle biopsy in the same institution. The accuracy was analyzed with the pathology as the gold standard. App participants were women and the mean age was 46.1 ± 11.23 years (range, 20–87 years).

## Results

### Diagnostic performance of quantitative parameters

Of all lesions, 334 (74.2%) were benign and 116 (25.8%) were malignant. The mean size of the breast masses was 1.18 ± 0.8 cm for the benign lesions and 1.67 ± 1.06 cm for the malignant lesions. The diagnostic performance of SWE and SMI quantitative parameters for distinguishing between benign and malignant breast masses is summarized in Table 1. The optimal cutoff values were 52.25 kPa for SWEmax, 5.03 for SWEratio, and 2.15% for SMI_VI_, with AUROC values of 0.881, 0.850, and 0.817, respectively.

**Table 1:** Diagnostic performance of quantitative parameters of SWE and SMI.

Variables	Cut off*	Sensitivity (%)	Specificity (%)	Accuracy (%)	PPV (%)	NPV (%)	AUROC (95% CI)
SWE_max_ (kPa)	52.25	79.3 (91/116)	90.1 (305/338)	87.3 (396/454)	73.6 (91/124)	92.6 (305/330)	0.881 (0.843–0.921)
SWE_ratio_ (%)	5.03	82.8 (96/116)	80.8 (272/338)	81.3 (368/454)	60.0 (96/162)	93.1 (272/292)	0.850 (0.811–0.884)
SMI_VI_ (%)	2.15	85.3 (99/116)	68.3 (229/338)	72.7 (328/454)	48.3 (99/208)	93.1 (229/246)	0.817 (0.790–0.851)

Data are percentages with the number of malignancies or the number of lesions in parentheses.

PPV: Positive predictive value, NPV: Negative predictive value, AUROC: Area under the receiver operating characteristics curve, CI: Confidence interval.

*The optimal cut off values for SWEmax, SWEratio and SMI_**VI**_ were determined based on the Youden’s index.

### Comparison of diagnostic performance between BI-RADS alone and combined scores

**Figure 2:**
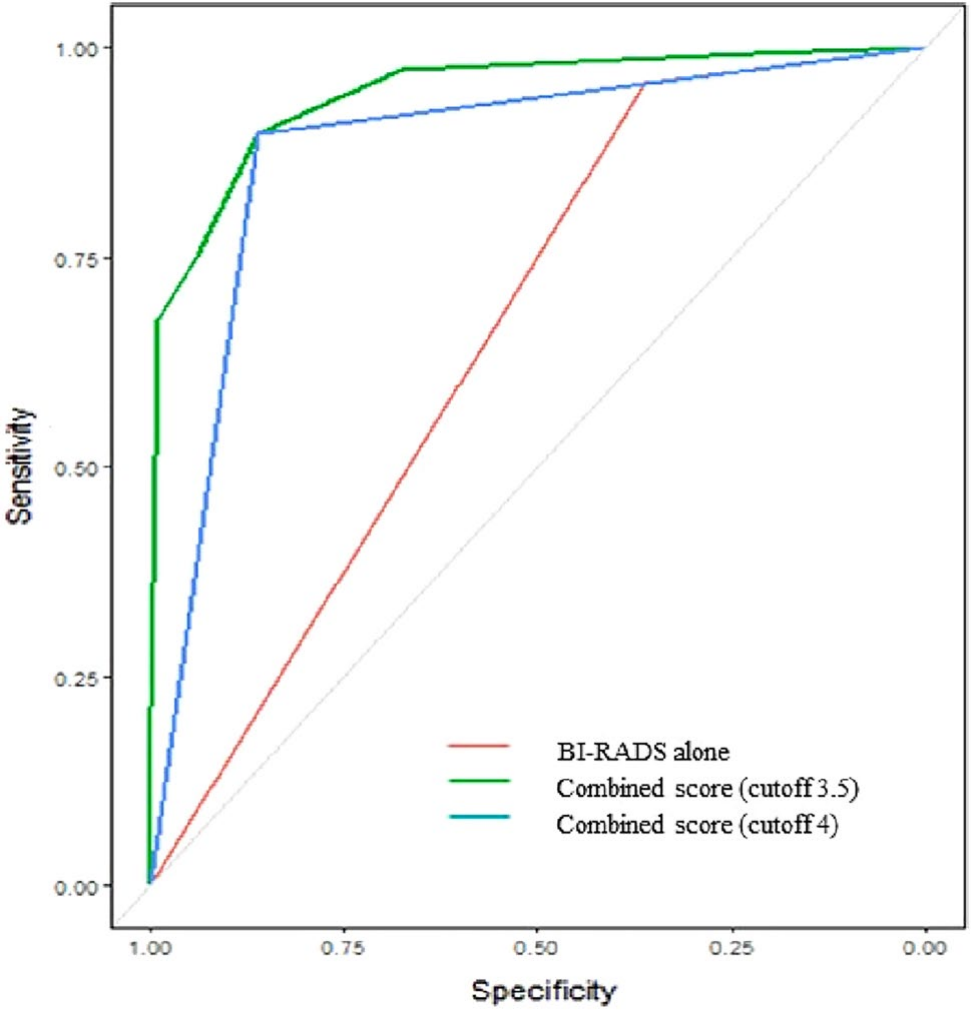
Receiver operator characteristics (ROC) curve analysis showed a statistically significant increase in the AUC value of combined score compared to BI-RADS alone.

**Figure 3:**
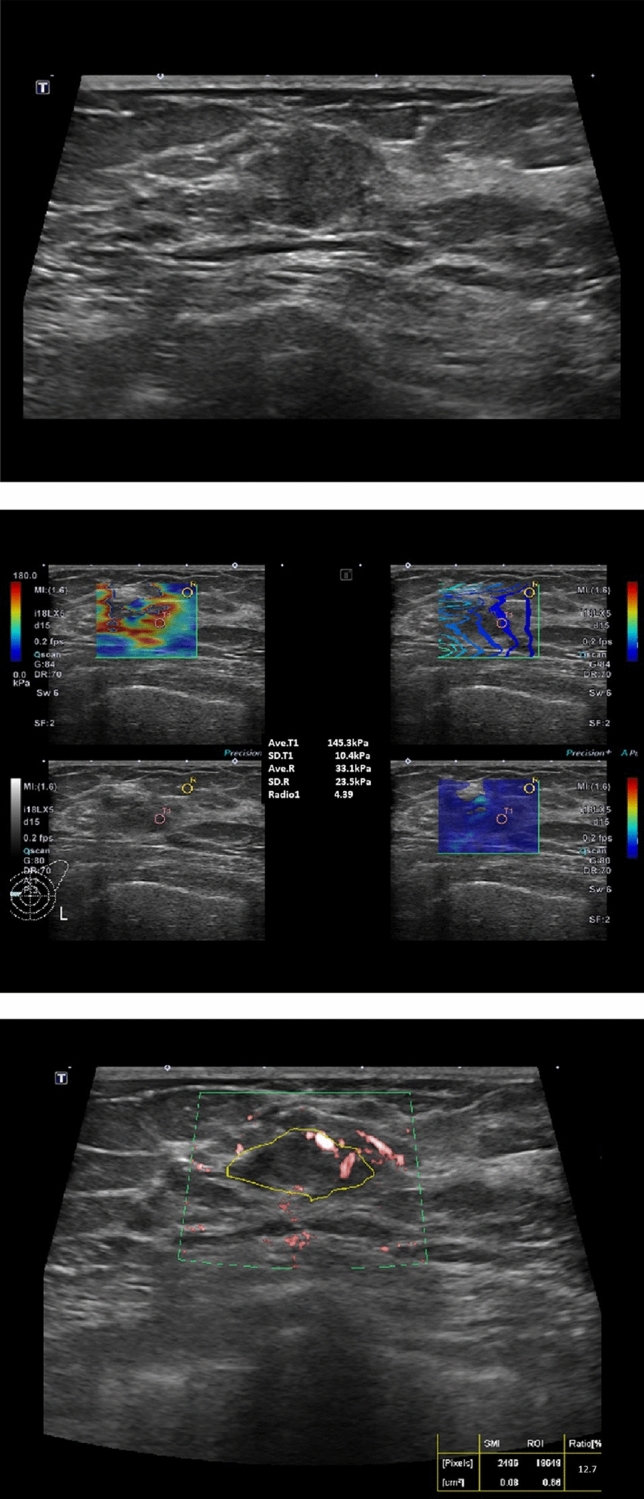
A 79-year-old woman with proven invasive ductal carcinoma. (**A**) B-mode ultrasound was a 1.5 cm-sized mass categorized as C4a. (**B**) The SWEmax was 145.3 kPa and the SWE ratio was 4.39. (**C**) The SMI vascular index was 12.7% over the cutoff value. The combined score was 4, indicating that the lesion was considered malignant lesion and the pathologic diagnosis was IDC grade I with DCIS.

When the combined scores were compared to B-mode only, the combined scores showed significantly higher AUROC values than BI-RADS alone (0.947 vs. 0.663, *p* < 0.001). Although the cut-off value of the combined score was 3.5, the cut-off value 4 was applied using round-off. The combined scores with a cutoff value of 4 showed significantly better diagnostic performance compared to BI-RADS only (Fig. 2). Compared to BI-RADS alone, the combined scores showed significant increases in specificity (36.8% vs. 86.5%, *p* < 0.001), accuracy (52.0% vs. 87.3%, *p* < 0.001), and PPV (34.5% vs. 69.8%, *p* < 0.001) with no statistically significant loss of sensitivity (95.7% vs. 89.7%, *p* = 0.085) (Table 2, Fig. 3).

**Table 2:** Comparison of diagnostic performance between BI-RADS alone and combined BI-RADS, SWEmax, SWEratio and SMI_**VI**_ scores.

Variables	Cut off	Sensitivity (%)	Specificity (%)	Accuracy (%)	PPV (%)	NPV (%)	AUROC (95% CI)
BIRADS (C3-C5)^a^ Combination score (1–8)^b^	4a	95.7 (111/116)	36.8 (123/334)	52.0 (234/450)	34.5 (111/322)	96.1 (123/128)	**0.663 (0.635–0.694)**
	3.5	89.7 (104/116)	86.5 (289/334)	87.3 (393/450)	69.8 (104/149)	96.0 (289/301)	**0.947 (0.922–0.968)**
	4	89.7 (104/116)	86.5 (289/338)	87.3 (393/450)	69.8 (104/149)	96.0 (289/301)	**0.877 (0.852–0.905)**
*p*-value ^c)^		0.085	< 0.001	< 0.001	< 0.001	0.747	< 0.001

		Sensitivity (%) (95% CI)	Specificity (%) (95% CI)	Accuracy (%) (95% CI)	PPV (%) (95% CI)	NPV (%) (95% CI)	AUROC (95% CI)
Validation for combination score (n = 524)	4	89.1 (81.4–94.4)	91.9 (88.9–94.4)	91.4 (88.7–93.7)	72.6 (63.9–80.2)	97.3 (95.1–98.6)	0.905 (0.872–0.939)

Significant values are in bold.

^a^For the evaluation of the diagnostic performance of B-mode ultrasound alone, BI-RADS categories were benign for category 3 and malignant for category 4a or higher.

^b^To calculate the combination score, BI-RADS categories were scored 1 to 5 (C3: 1, C4a: 2, C4b: 3, C4c: 4, and C5: 5) and quantitative parameters of SWE and SMI were scored 0 or 1 according to each cutoff value and ranged from 1 to 8 score.

^c^Diagnostic performances of B-mode alone and combined score were compared. PPV: Positive predictive value, NPV: Negative predictive value, AUROC: Area under the receiver operating characteristics curve, CI: Confidence interval.

### Analysis of BI-RADS category and combined score according to pathology

**Figure 4:**
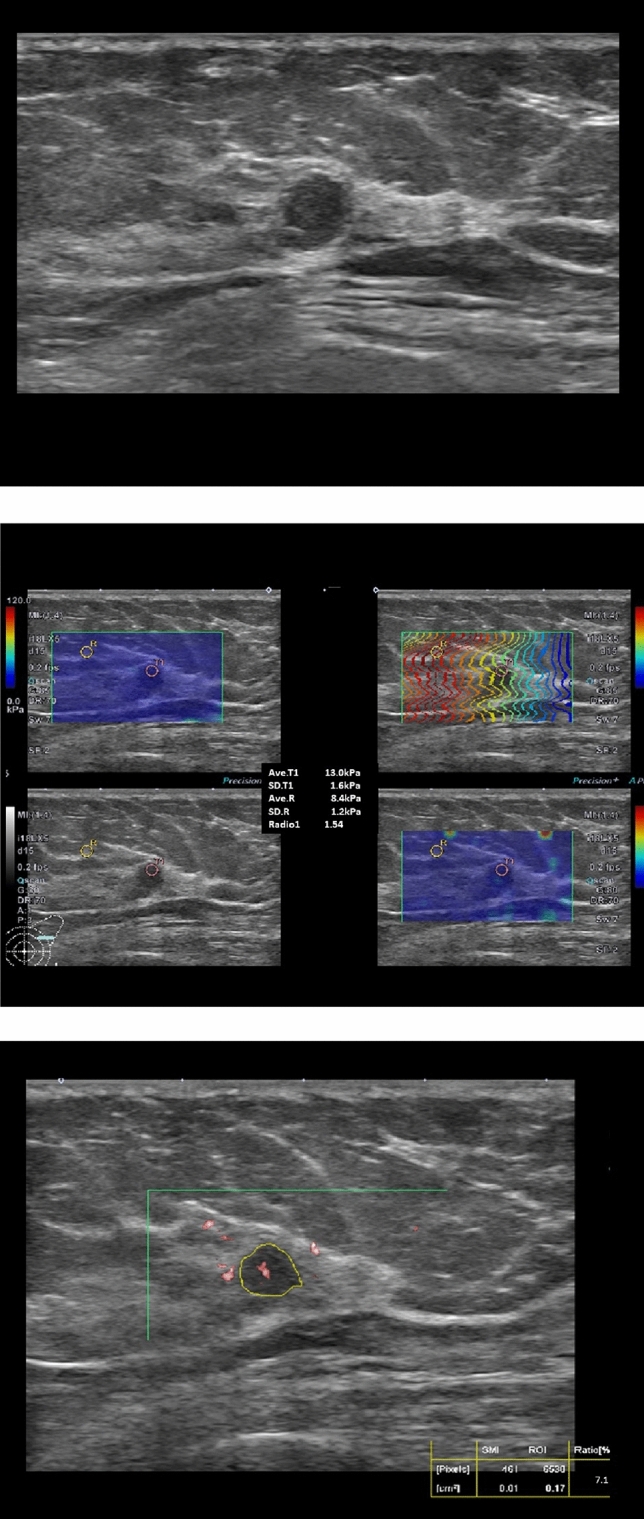
DCIS in a 50-year-old female patient. (**A**) B-mode ultrasound showed a 0.6 m-sized, mass categorized as C4a. (**B**) The lesion had soft elasticity measured below the cutoff values as SWEmax 13.0 kPa and SWEratio 1.54. (**C**) The SMI vascular index was measured at 7.1%, over the cutoff value. The combined score was 3, representing a benign lesion, but the pathologic diagnosis was DCIS, intermediate.

There were 12 malignant cases with combined scores of 1 to 3, representing false negative rate of 2.7% (12/450). Among the two cases of C3 lesions, one case of malignant phyllodes tumor showed a combined score of 3, which was over the SWEmax and SWEratio cutoff values and less than the SMI_VI_ cutoff value. The other C3 lesion showed all quantitative parameters less than the cutoff value but was pathologically diagnosed as invasive ductal carcinoma with necrosis. The 10 cases of false negative C4a with combined scores below 4 were DCIS (n = 5), IDC grade I (n = 4) and ILC (n = 1), with a mean size of 0.68 cm (Fig. 4). Of the 123 cases of C3 lesions considered benign by a combined score of 1 to 3, two cases were pathologically malignant, and false negative rate of C3 lesion were 1.6% (2/123).

**Figure 5:**
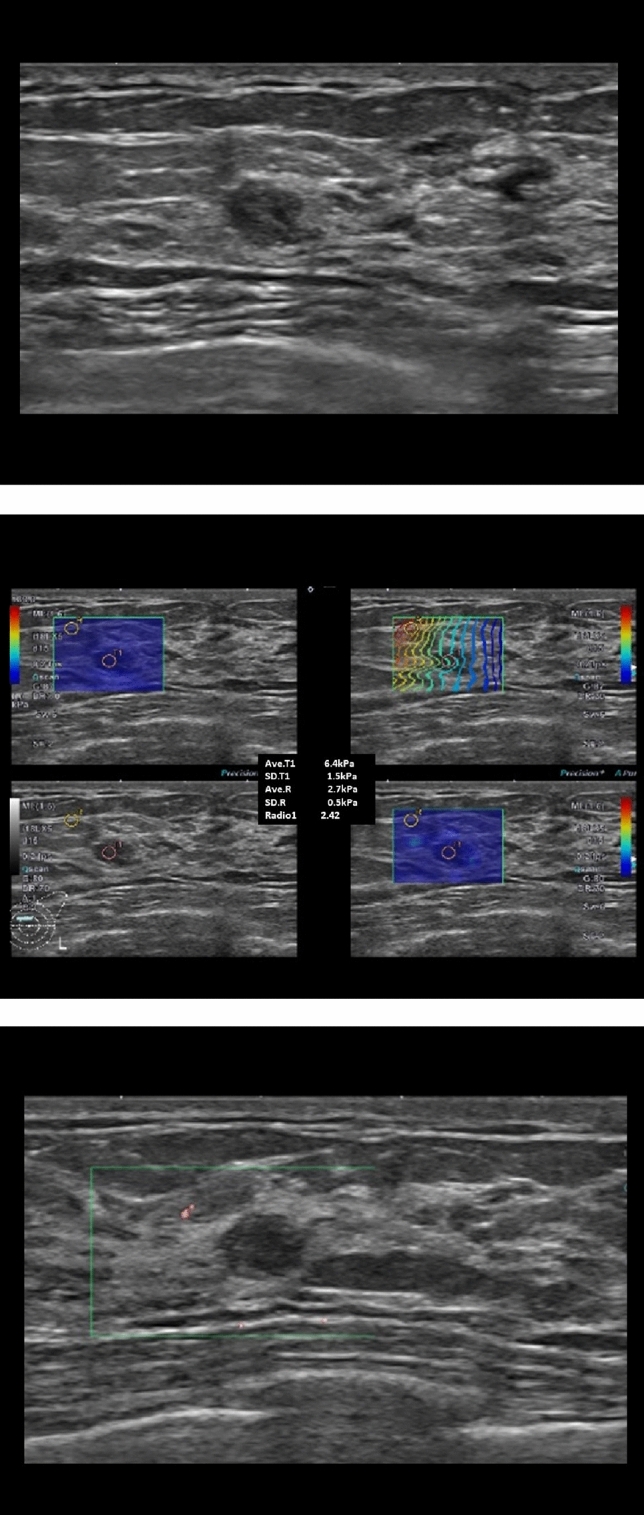
A 32-year-old female patient with fibroadenoma. (**A**) B-mode ultrasound showed a 0.6 cm-sized, mass categorized as C4a. (**B**) The SWEmax and SWE ratio were below the cutoff value as as SWEmax 6.4 kPa and SWEratio 2.42. (**C**) The vascular index of the SMI was 0%. The combined score was 2, indicating that the lesion was considered benign lesion. The pathologic diagnosis was fibroadenoma.

Among 104 malignant cases with combined scores of over 4, three cases were categorized as C3, but the combined score was 4 because all quantitative parameters were above the cutoff values. The pathologically diagnoses were lymphoma (n = 1), DCIS (n = 1), and IDC (n = 1). Forty-five lesions (10.0%, 45/450) were false positive which were pathologically benign but showed combined scores over 4 including two C3 lesions and 43 lesions classified as C4a to C4c. In 216 cases of C4a lesions, 165 cases (76.4%, 165/216) were considered benign when applying the combined scores and the pathologic diagnosis was also benign (Table 3, Fig. 5).

**Table 3:** Comparison of BI-RADS category and combined score between benign and malignant masses according to pathology.

BIRADS total 450 (100)	Benign 334 (74.2)		Malignant 116 (25.8)	
	True negative 289 (64.2) (combined score 1–3)	False positive 45 (10.0) (combined score 4–8)	False negative 12 (2.7) (combined score 1–3)	True positive 104 (23.1) (combined score 4–8)
C3 128 (28.4)	121	2	2	3
C4a 216 (48.0)	165	28	10	13
C4b 32 (7.1)	3	11	0	18
C4c 45 (10.0)	0	4	0	41
C5 29 (6.4)	0	0	0	29
Pathology	C3: Fibrocystic change 59, Fibroadenoma 56, IDP 3, Phyllodes (benign) 3 C4a: Fibrocystic change 93, Fibroadenoma 51, IDP 10, Phyllodes (benign) 6, Sclerosing adenosis 3, PASH 2 C4b: Fibrocystic change3	C3: Fibrocystic change 2 C4a: Fibrocystic change 6, Fibroadenoma 11, IDP 7, Phyllodes (benign) 3, Sclerosing adenosis 1 C4b: Fibrocystic change 5, IDP 5, Abscess 1 C4c: Fibrocystic change 3, Fibroadenoma 1	C3: Phyllodes (malingnant)1, IDC 1 C4a: DCIS 5, IDC 4, ILC 1	C3: Lymphoma 1, DCIS 1, IDC 1 C4a: IDC 11, DCIS 2 C4b: IDC 17, ILC 1 C4c: IDC 31, ILC 3, DCIS 7 C5: IDC 26, ILC 1, DCIS 2

Values are presented as number (%).

IDP: intraductal papilloma, PASH: pseudoangiomatous stromal hyperplasia, DCIS: ductal carcinoma in situ, IDC: infiltrative ductal carcinoma, ILC: infiltrative lobular carcinoma. BI-RADS category 3: C3, category 4a:C4a, category 4b: C4b, category 5:C5.

### Validation analysis of combined score according to pathology

Of all validation lesions, 423 (80.7%) were benign and 101 (19.3%) were malignant. The mean size of the breast masses were 1.17 ± 0.7 cm for the benign lesions and 1.70 ± 1.06 cm for the malignant lesions, When the cut-off value 4 was applied for validation patients, the accuracy was 91.4% (88.7–93.7, 95% confidence interval) (Table 2).

## Discussion

In this prospective study, we evaluated the diagnostic performance of combined scores divided as the sum of the BI-RADS score and the quantitative value scores. All parameters were scored as BI-RADS categories C3 to C5 using one to five points to reflect the weight of each category, and each quantitative value was dichotomized based on each cutoff value. The combined scores showed significant better diagnostic performance with 86.5% specificity and 87.3% accuracy without a statistically significant loss of sensitivity compared to B-mode US alone. According to a meta-analysis study of adding SWE to B-mode US for the detection of breast cancer, SWE with B-mode US significantly improved specificity in differentiating between benign and malignant lesions and reduces the unnecessary biopsies of the patient having benign lesions^7^. A study on the combined use of the SMI vascular index and B-mode US, a recently introduced quantitative parameter, also showed improved diagnostic performance in distinguishing benign and malignant breast lesions compared to B-mode alone, without a significant change in sensitivity^15,16^.

In our study, the SWE cutoff value was 52.25 kPa for SWEmax, 5.03 for SWEratio, and 2.15% for SMI_VI_, which were within the previously reported ranges. However, a previous reported retrospective study showed cutoff values of 86.45 kPa for SWEmax, 3.57 for SWEratio, and 3.35% for SMI_VI_^18^., which were different ranges than in this study. The difference in cutoff values in this study was likely related to the fact that large numbers of patients who underwent a core needle biopsy were prospectively enrolled and had a relatively high percentage of C4a lesions (48.0%, 216/450), and many of these lesions were benign (89.4%, 193/216). There is no standardized cutoff value for SWE, and the cutoff values in previous studies varied over a wide range of 45.1–124.9 kPa for Emax and 3.56–5.14 for SWEratio^7,9,20–24^. The previously reported cutoff values of SMI_VI_ ranged from 2.95 to 8.9%^4,15,16,18,25^, and were not measured in real-time US in most of the studies because the SMI_VI_ measurements were obtained using post-processing software of the acquired images^4,15,25^. The relatively wide range of cutoff values is probably due to the characteristics of breast lesions such as lesion size, the histological malignancy type, overlapping features between benign and malignant lesions. There is also probably related with various study populations, US equipment and methodology of measurement in the reported studies^7,26^.

The benefit of reducing unnecessary biopsies by adding the SWE or SMI quantitative parameters must be supported by a sufficiently low false-negative rate. According to a previous study, when SWE was combined with B-mode US, the frequency of unnecessary biopsies decreased by 71.3%, and the false-negative rate was a mean of 3.1% (range, 0–9.4%)^2,7,27–31^. A review of previous studies, found that applying lower cutoff values such as < 40 kPa might be an important strategy to decrease the prevalence of false-negative cases^2,7,27^. When downgrading BI-RADS C4a lesions to C3, the false negative rate was 6.6% when an Emax cutoff of 87.5 kPa was used, but the false negative rate decreased to 0% when an Emax cutoff of 50 kPa was used^27^. Studies using Emax cutoff of 145.9 kPa reported high false negative rates of 8.0–9.4%^31^. In our study, when the combined score was used, the frequency of unnecessary biopsies decreased by 76.4% (165/216), and the false-negative rate was low at 2.7% (12/450). Relatively low elasticity values in SWE were seen in soft malignant lesions such as DCIS, low-grade IDC, lobular carcinoma, mucinous carcinoma and lymphoma, small-sized malignancies, and lesion located in deep portion^8,18,27,28,30,32,33^. Some benign lesions, such as fat necrosis and mastitis, have relatively high elasticity values^7,8^. Of the 12 false negative cases in our study population, 10 cases were category 4a and the pathologic results were DCIS (n = 5), IDC grade I (n = 4) and ILC (n = 1), with a mean lesion size of 0.68 cm, consistent with previous studies. Of the C3 cases, 1.1% (5/450) were upgraded by a combined score of 4 or more, suggesting malignant potential. Three cases were malignancies such as lymphoma, DCIS, and IDC, and two cases were benign fibrocystic change.

For the appropriate clinical application of SWE and SMI quantitative parameters, radiologists should evaluate and monitor the optimal cutoff value for each institution because the type of equipment used, the experience of the operator, and the characteristics of the lesion may affect the measurements of the quantitative parameters. In addition, combined scores should be carefully applied considering the quantitative parameters have limitations in assessing small-sized, pure DCIS, or low-grade invasive cancer. The radiologists carefully need to decide whether to perform a biopsy considering the relatively low elasticity of some malignant lesions, even if the combined score is below the cut value 4 for C4a lesions. If two of the three quantitative parameters is over the cut-off value in C3 lesion even if the combined score is below the cut-value 4, a careful short follow -up may be required.

There were several limitations to our study. First, since this was a small single-institution study, there is a limitation in generalizing the optimal cutoff value and these cut off values may be affected by equipment used or operator’s experience could influence the quantitative parameters. Second, B-mode US, SWE, and SMI were performed simultaneously by the same radiologist, and each result may have influenced the other in BI-RADS categorization or selection of representative SWE and SMI measurements. Third, many of patients were underwent US-guided core needle biopsy after receiving B-mode and quantitative parameters at the same day, which may have influenced the assignment of the BI-RADS category. In addition, although each lesion can be directly compared with pathological result by performing core needle biopsy after B-mode and quantitative parameters, there cannot be also excluded the possibility of pathological under-estimation by core needle biopsy. Further studies are needed with an independent set of patients testing for the combined score. For the validation, when applying the combination score to which the cutoff value was applied to patients from a single institution, it showed a high accuracy of 91.4%. However, to find the clinically acceptable optimal cutoff values and for investigation the effects of adding the SWE and SMI quantitative parameters to those of the B-mode in patient management in actual clinical practice, further studies for the multicenter large populations are needed.

In conclusion, combined B-mode US and SWEmax, SWEratio, and SMI_VI_ quantitative parameter scores improved the diagnostic performance in differentiating between benign and malignant breast masses. Combined scoring could be helpful in determining the need for a breast biopsy if applied carefully.

## Abbreviations

SWE: Shear wave elastography
SWEmax: Maximum elasticity of shear wave elastography
SWEratio: Elasticity ratio between the lesion and subcutaneous fat tissue
SMI: Superb microvascular image
SMI_VI_: Vascular index of superb microvascular image
US: Ultrasonography
BI-RADS: Breast imaging reporting and data system
ROI: Region of interest
AUROC: Area under the receiver operating characteristics curve
PPV: Positive predictive value
NPV: Negative predictive value
DCIS: Ductal carcinoma in situ
IDC: Infiltrative ductal carcinoma
ILC: Infiltrative lobular carcinoma
